# Awareness of Blindness Related to Smoking Among Young Age Population: A Cross-Sectional Study in Saudi Arabia

**DOI:** 10.7759/cureus.30501

**Published:** 2022-10-20

**Authors:** Maryam O Alarfaj, Munif M Alshammari, Hanan A Albenayyan, Amar A Alonazi, Abdullah A Alkhateeb, Abdulaziz Al Taisan

**Affiliations:** 1 Medicine, King Faisal University, Hofuf, SAU; 2 Medicine, King Faisal University, Khafji, SAU; 3 Medicine, King Faisal University, Dammam, SAU; 4 Medicine, King Faisal University, Hufof, SAU; 5 Medicine, King Faisal University, Al-Ahsa, SAU

**Keywords:** awareness, saudi arabia, young population, blindness, smoking

## Abstract

Globally, it is known that smoking can cause a variety of diseases. Studies have shown that smoking not only causes heart and lung diseases but is also strongly related to ocular diseases which could lead to blindness. This study aims to assess the level of awareness of blindness related to smoking in young people in Saudi Arabia.

This is a descriptive cross-sectional study conducted among the young population in Saudi Arabia. A self-administered questionnaire was distributed using an online platform that includes socio-demographic data, education level, smoking status, and knowledge of the harmful effect of smoking like lung and cardiac diseases, including blindness. The data were analyzed using Statistical Packages for Social Sciences (SPSS) version 26 (IBM Corp., Armonk, NY).

A total of 655 young individuals responded to the survey. Among our subjects, the prevalence of smoking participants was (18.7%) more common among the age group 21-25 years, males, bachelor, or higher degrees and those living in the Central region (p<0.001). Participants were definitely or probably more aware that the most common condition associated with excessive smoking was lung cancer (99.5%), followed by heart disease (98.1%) and stroke (93%) while the least of them was melanoma (91.3%) and blindness (81%).

This is the first study in Saudi Arabia to assess the level of awareness of the risks of blindness related to smoking in young people and disclosed limited knowledge of the effect of smoking on their vision. The effects of smoking on lung cancer, heart disease, stroke, and even melanoma received higher ratings than blindness. Smokers may be able to quit smoking and enhance their quality of life by better understanding the link between smoking and blindness, which suggests that appropriate action is required to enhance their awareness.

## Introduction

Smoking is the leading preventable cause of many diseases and deaths worldwide. It is an important risk factor for diseases such as cardiovascular and pulmonary diseases and different types of cancer. Although a majority of smokers are aware of the increased risk of premature death due to smoking, they are less likely aware of its harmful effects in causing disability and a reduction in quality of life [1]. In addition, studies have shown that smoking is strongly related to ocular diseases, including age-related macular degeneration, cataract, Graves’ ophthalmopathy, and glaucoma [2]. Age-related macular degeneration and cataract are the leading causes of blindness worldwide [3,4]. More importantly, although many people are aware of the association between smoking and diseases such as lung cancer, heart disease, and stroke, only a few are aware of its association with blindness [5,6].^ ^In a cross-sectional study conducted at General Hospital in the United Kingdom between May and June 2004, only 12% of eye clinic patients and 7% of other clinic attendees believed smoking causes blindness. However, 70%, >85%, and >90% believed that smoking causes stroke, heart disease, and lung cancer, respectively [7]. Another study conducted in Canada, the United States, the United Kingdom, and Australia between 2004 and 2007 found that the knowledge that smoking could cause blindness varies across countries; 13.0% in Canada, 9.5% in the United States, 9.7% in the United Kingdom, and 47.2% in Australia believed that smoking causes blindness [8]. In Saudi Arabia, the number of smokers is continuously increasing. According to two studies conducted in 2013 and 2018, the prevalence of smoking among the Saudi population increased from 12.2% in 2013 to 21.4% in 2018 [9,10]. Furthermore, according to a systematic review and meta-analysis conducted between 2010 and 2018, the prevalence of smoking among Saudi college students was higher (17%) than in most regional countries [11]. According to a study conducted among university students in Abha, Saudi Arabia, only 34.8% of the participants were aware of the impact of smoking on diabetic retinopathy progression [12]. However, there are limited studies investigating the association between smoking and blindness in Saudi Arabia. This study aimed to determine the awareness level among the young Saudi population of the association between smoking and blindness.

## Materials and methods

This is a descriptive cross-sectional study conducted among the young population of Saudi Arabia. A self-administered questionnaire, including socio-demographic data, education level, and smoking status, was distributed using an online platform. It also investigated the awareness and fear of blindness for three established smoking-related diseases, lung and heart diseases, blindness and melanoma (a distractor condition), and the likelihood that smokers would quit on developing early signs of each condition. We added the distractor factor to decrease the number of over-reporting questions related to blindness.

The calculated sample size was 382 young participants. The following statistical formula was used to calculate the representative sample size based on the study population (confidence interval [CI], 95%; margin of error, 5%):

Young male and female participants aged 10 to 34 years from all regions of Saudi Arabia were included in the selection criteria. Participants <10 years or >34 Research years of age, anyone outside Saudi Arabia and those who fail to complete the questionnaire were excluded from the study. Ethical approval was obtained by the Institutional Research Board and the Ethics Committee of King Faisal University in Al-Ahsa after fulfilling all the needed ethical issues (Research Number: KFU-REC-2021-DEC-EA000259).

Categorical variables were summarized as frequencies and proportions (%). Continuous variables were presented as means and standard deviations. For comparison, the Chi-square test (categorical variables) and Mann-Whitney U test (continuous variables) were applied. Normality tests were performed using the Shapiro-Wilk test. A p-value of 0.05 at 95% CI was considered statistically significant. Data were analyzed using Statistical Packages for Social Sciences (SPSS) version 26 (IBM Corp., Armonk, NY).

## Results

The participants’ socio-demographic characteristics in accordance with their smoking status are shown in Table 1.

**Table 1:** Socio-demographic characteristics of participants according to smoking status

Study Data	Overall N (%) (n=589)	Smoking status		P-value ^§^
		Non-smoker N (%) (n=479)	Smoker N (%) (n=10)	
Age group				
≤20 years	208 (36.3%)	193 (40.3%)	15 (13.6%)	<0.001 **
21 – 25 years	241 (40.9%)	185 (38.6%)	56 (50.9%)	
>25 years	140 (23.8%)	101 (21.1%)	39 (35.5%)	
Gender				
Male	184 (31.2%)	118 (24.6%)	66 (60.0%)	<0.001 **
Female	405 (68.8%)	361 (75.4%)	44 (40.0%)	
Educational level				
Diploma or below	176 (29.9%)	152 (31.7%)	24 (21.8%)	0.040 **
Bachelor or higher	413 (70.1%)	327 (68.3%)	86 (78.2%)	
Residence region				
Eastern region	128 (21.7%)	109 (22.8%)	19 (17.3%)	<0.001 **
Central region	86 (14.6%)	51 (10.6%)	35 (31.8%)	
Western region	126 (21.4%)	114 (23.8%)	12 (10.9%)	
Northern region	140 (23.8%)	112 (23.4%)	28 (25.5%)	
Southern region	109 (18.5%)	93 (19.4%)	16 (14.5%)	

In this study, the most common age group was 21-25 years (40.9%), with females being more dominant (68.8%) than males. Regarding the participants’ education, the majority held a bachelor’s degree or higher (70.1%). Regarding residence location, 23.8% lived in the Northern region, while 21.7% lived in the Eastern region. Furthermore, the prevalence of smoking was 18.7%, and it was more common among the age group 21-25 years (p<0.001), males (p<0.001), bachelor or higher degree holders (p<0.001), and those living in the Central region of Saudi Arabia (p<0.001). Regarding the participants’ smoking behavior, 55.5% smoked one packet of cigarettes per day; of them, the smoking duration ranged from one to five years in 35.5%. Table 2 suggests that 80%, 80%, 80%, 75.5%, and 72.7% of the participants would quit smoking if they developed early signs of lung cancer, heart disease, stroke, blindness, and melanoma, respectively.

**Table 2:** Behavior of smoking participants toward its risk factors (n=110)

Variables	N (%)
How many cigarettes per day?	
Less than 1 packet/day	61 (55.5%)
1 - 2 packets/day	38 (34.5%)
>2 packets/day	11 (10.0%)
Years of smoking	
Less than 1 year	30 (27.3%)
1 - 5 years	39 (35.5%)
6 - 10 years	27 (24.5%)
11 - 20 years	14 (12.7%)
Would you quit smoking if you developed early signs of lung cancer?	
Yes	88 (80.0%)
No	04 (03.6%)
Maybe	18 (16.4%)
Would you quit smoking if you developed early signs of heart disease?	
Yes	88 (80.0%)
No	04 (03.6%)
Maybe	18 (16.4%)
Would you quit smoking if you developed early signs of stroke?	
Yes	88 (80.0%)
No	03 (02.7%)
Maybe	19 (17.3%)
Would you quit smoking if you developed early signs of blindness?	
Yes	83 (75.5%)
No	07 (06.4%)
Maybe	20 (18.2%)
Would you quit smoking if you developed early signs of melanoma?	
Yes	80 (72.7%)
No	08 (07.3%)
Maybe	22 (20.0%)

**Figure 1:**
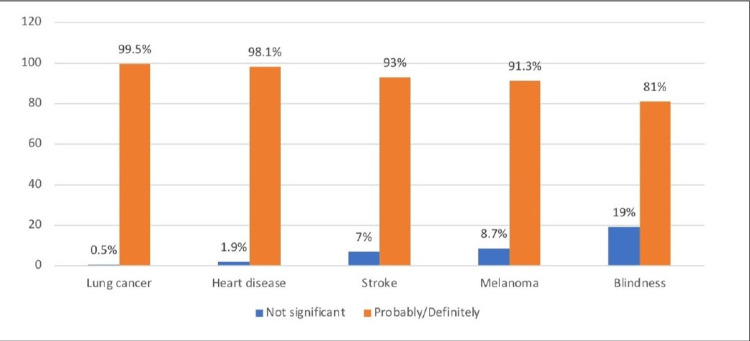
Knowledge about the condition caused by smoking

**Figure 2:**
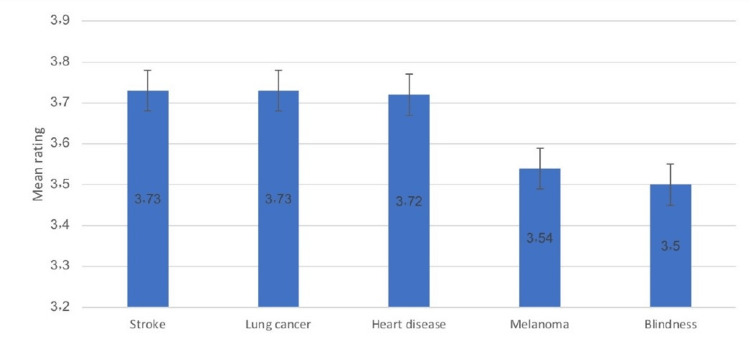
Ratings for the least to the most feared condition as a result of smoking (rank 1-5)

The participants were definitely or probably aware that the most common condition associated with excessive smoking was lung cancer (99.5%), followed by heart disease (98.1%), and stroke (93%), whereas the least associated conditions were melanoma (91.3%) and blindness (81%) (Figure 1). Figure 2 indicates that stroke (mean score: 3.73), lung cancer (mean score: 3.73), and heart disease (mean score: 3.72) were the most feared conditions associated with smoking, whereas melanoma (mean score: 3.54) and blindness (mean score: 3.5) were the least feared conditions.

One hundred and nine smoking participants believed that smoking would definitely or could probably cause lung cancer, 108 believed that it could cause heart disease, 100 indicated strokes, 97 indicated melanoma, and 86 indicated blindness (Table 3).

**Table 3:** Proportion of participants who believe that smoking definitely or probably caused conditions

Condition	Smoking status	
	Non-smoker N (%) ^(n=479)^	Smoker N (%) ^(n=110)^
Lung cancer	477	109
Heart disease	470	108
Stroke	448	100
Blindness	391	86
Melanoma	441	97

When comparing the ratings for the least or most feared condition between smoking and non-smoking participants, the results revealed that non-smoking participants have higher ratings of fear that smoking could cause lung cancer (p=0.030), heart disease (p=0.019), stroke (p=0.022), blindness (p=0.010), and melanoma (p<0.001) (see Table 4). When measuring beliefs on whether or not smoking can cause blindness, according to the participants’ smoking status and demographic data, we found that the number of male participants who believed smoking could “probably” or “definitely” cause blindness was statistically significantly higher than female participants (p=0.008), while the differences in age group (p=0.882), an education level (p=0.324), and residence region (p=0.260) did not reach statistical significance (see Table 5).

**Table 4:** Rank ratings for the least or most feared condition between smoker and non-smoker (n=589)

Condition	Smoking status		P-value ^§^
	Non-smoker Mean ± SD	Smoker Mean ± SD	
Lung cancer	3.81 ± 1.64	3.37 ± 1.79	0.030 **
Heart disease	3.80 ± 1.49	3.35 ± 1.68	0.019 **
Stroke	3.85 ± 1.51	3.37 ± 1.74	0.022 **
Blindness	3.60 ± 1.44	3.09 ± 1.69	0.010 **
Melanoma	3.67 ± 1.51	2.98 ± 1.73	<0.001 **

**Table 5:** Relationship between believing that smoking definitely or probably caused blindness and the socio-demographic characteristics of smoking participants (n=110)

Factor	Smoking caused blindness		P-value
	Probably/Definitely N (%) (n=86)	Not significant N (%) (n=24)	
Age group			
≤20 years	11 (12.8%)	04 (16.7%)	0.882
21 – 25 years	44 (51.2%)	12 (50.0%)	
>25 years	31 (36.0%)	08 (33.3%)	
Gender			
Male	46 (53.5%)	20 (83.3%)	0.008 **
Female	40 (46.5%)	04 (16.7%)	
Educational level			
Diploma or below	17 (19.8%)	07 (29.2%)	0.324
Bachelor or higher	69 (80.2%)	17 (70.8%)	
Residence region			
Eastern region	16 (18.6%)	03 (12.5%)	0.260
Central region	31 (36.0%)	04 (16.7%)	
Western region	09 (10.5%)	03 (12.5%)	
Northern region	19 (22.1%)	09 (37.5%)	
Southern region	11 (12.8%)	05 (20.8%)	

## Discussion

This study attempts to evaluate the awareness of blindness related to smoking among the young age population. To our knowledge, this is the first study in Saudi Arabia to have examined the knowledge of the young population regarding the link between smoking and blindness. Findings of this showed that 81% of the smokers and non-smokers thought that smoking can definitely or probably can cause blindness lower than the awareness they had about other conditions associated with smoking such as lung cancer (99.5%), heart disease (98.1%) and stroke (93%). Interestingly, 91.3% of the sample population rated the distractor variable, melanoma as a complication of smoking, with higher ratings than blindness. This indicates the need for awareness campaigns to educate young people about the specific diseases associated with smoking. A study performed by Kennedy et al. [8] documented that the knowledge of smoking causes blindness was higher among the adult population in Australia (47.2%), however, fewer people from Canada (13%), the USA (9.5%), and UK (9.7%) believed that blindness is a complication of smoking. Respondents further believed that secondhand smoke was the most dangerous one as it may cause cancer (77%) and stroke (80.1%). Consistent with these findings, Handa et al. [13] indicated that 42.5% of the eye patients were aware of the link between smoking and irreversible blindness but they had shown better awareness about the association between smoking and lung cancer (85%) comparable to the reports published by Bidwell et al. [14] as well as Moradi et al. [15]. However, in a cross-sectional study by Dawood et al. [16], Iraqi smokers showed poor awareness regarding some risk factors of smoking such as lung cancer in non-smokers (30.1%), impotence in male smokers (52.6%), premature aging (64%) and stroke (66.3%) adding that extensive efforts are needed to raise awareness about the risk of smoking and health benefits of smoking cessation.

There have been many reports that lung cancer, heart disease, and stroke were the most common complications resulting from smoking. However, blindness due to smoking seems to be not being recognized by the population such that even in some of the first world countries like Canada, the USA, and the UK, smokers are less likely to know that smoking causes blindness [8]. In our study, male smokers are more likely to know that smoking can definitely or probably cause blindness. In Singapore [13], however, there was no association between awareness of smoking-related blindness among gender and races, but authors noted a better awareness about the link between smoking among heart attack, stroke, and other lung diseases could be more associated with a better knowledge of smoking-related blindness.

Data in our study also suggest that stroke (mean: 3.73), lung cancer (mean:3.73) and heart disease (mean: 3.72) were the most feared complications of smoking but they exhibited less fear of blindness (mean: 3.5) while the distractor disease (melanoma) has a mean of 3.54 with non-smokers were more feared of having all these conditions than smokers were. These findings seemed to be not following the report of Moradi and colleagues [15] where they found out that the patients were more fearful of blindness than of lung cancer, heart disease, and deafness. On the contrary, Bidwell et al. [16] reported that adult patients were less likely to be fearful of blindness than lung cancer, heart disease, and stroke. In a study conducted by Oncken et al. [1], the general population reported that the perceived risk of smoking could be more associated with premature death (95%) and there were 63.5% believed that smoking was also linked to disabilities with the knowledge and perceived risk of reproductive-related problems and cancers was much lower.

Pertaining to the perception of quitting, higher intention to quit was seen if the subjects developed lung cancer (80%), heart disease (80%), and stroke (80%) with slightly less in blindness (75.5%) and distractor variable melanoma (72.3%), while in our study, the population placed blindness as the fourth most intention reason to quit smoking; In a study done by Handa et al. [13], patients placed blindness as the second most motivating factor to quit smoking whereas lung cancer was the chief motivating factor. On the other hand, Bidwell et al. [14] reported that half of the smokers stated that they would definitely or probably quit smoking if they develop early signs of blindness followed by lung cancer, heart disease, and stroke. This corroborates the study conducted by Moradi et al. [15], who found out that fear of blindness may be more likely to motivate teenagers to stop smoking than fear of lung or heart disease with approximately 90% of teenagers would cease smoking if there was an early sign of blindness higher than lung (78%) or heart diseases (80%). Smoke cessation is not an easy task among smokers, Handa et al. [13] emphasized that smokers may be more inspired to quit smoking if they were permitted to do it slowly over some time rather than straight away. Consistent awareness about the hazardous effect of excessive smoking is necessary to avoid complications such as blindness, lung cancer, stroke, disability, and worst, premature death.

## Conclusions

The study demonstrates that young adults have limited knowledge of the effects of smoking on their vision. Awareness of the effects of smoking on lung cancer, heart disease, stroke, and even melanoma received higher ratings than blindness. In comparison to other conditions, young adults exhibited a lesser fear of blindness and a lower perception of quitting if they thought they would become blind. Smokers may be able to quit smoking and enhance their quality of life by better understanding the link between smoking and blindness. Healthcare workers, the media, and official authorities all have a role to play in informing and educating people about the risks of smoking-related blindness.
